# Neutrophil Protease Cleavage of Von Willebrand Factor in Glomeruli – An Anti-thrombotic Mechanism in the Kidney

**DOI:** 10.1016/j.ebiom.2017.01.032

**Published:** 2017-01-24

**Authors:** Ramesh Tati, Ann-Charlotte Kristoffersson, Minola Manea Hedström, Matthias Mörgelin, Jörgen Wieslander, Cees van Kooten, Diana Karpman

**Affiliations:** a Department of Pediatrics, Clinical Sciences Lund, Lund University, Lund, Sweden; b Department of Infection Medicine, Clinical Sciences Lund, Lund University, Lund, Sweden; c Department of Nephrology, Clinical Sciences Lund, Lund University, Lund, Sweden; d Department of Nephrology, Leiden University Medical Center, Leiden, The Netherlands.

**Keywords:** Von Willebrand factor, Glomerular basement membrane, Kidney, Neutrophils, ADAMTS13, Elastase

## Abstract

Adequate cleavage of von Willebrand factor (VWF) prevents formation of thrombi. ADAMTS13 is the main VWF-cleaving protease and its deficiency results in development of thrombotic microangiopathy. Besides ADAMTS13 other proteases may also possess VWF-cleaving activity, but their physiological importance in preventing thrombus formation is unknown. This study investigated if, and which, proteases could cleave VWF in the glomerulus. The content of the glomerular basement membrane (GBM) was studied as a reflection of processes occurring in the subendothelial glomerular space. VWF was incubated with human GBMs and VWF cleavage was assessed by multimer structure analysis, immunoblotting and mass spectrometry. VWF was cleaved into the smallest multimers by the GBM, which contained ADAMTS13 as well as neutrophil proteases, elastase, proteinase 3 (PR3), cathepsin-G and matrix-metalloproteinase 9. The most potent components of the GBM capable of VWF cleavage were in the serine protease or metalloprotease category, but not ADAMTS13. Neutralization of neutrophil serine proteases inhibited GBM-mediated VWF-cleaving activity, demonstrating a marked contribution of elastase and/or PR3. VWF-platelet strings formed on the surface of primary glomerular endothelial cells, in a perfusion system, were cleaved by both elastase and the GBM, a process blocked by elastase inhibitor. Ultramorphological studies of the human kidney demonstrated neutrophils releasing elastase into the GBM. Neutrophil proteases may contribute to VWF cleavage within the subendothelium, adjacent to the GBM, and thus regulate thrombus size. This anti-thrombotic mechanism would protect the normal kidney during inflammation and could also explain why most patients with ADAMTS13 deficiency do not develop severe kidney failure.

## Highlights

• Neutrophil proteases in the glomerular basement membrane cleave VWF and may protect the kidney from microthrombi.
• VWF cleavage would be activated by neutrophil influx and compensate for the prothrombotic mechanisms during inflammation.
• This mechanism may compensate for lack of ADAMTS13 and explain why TTP patients seldom develop end-stage renal failure.

The study demonstrates a mechanism by which the kidney is protected from blood clotting during inflammation. In the inflammatory setting white blood cells infiltrate tissues. In this study we showed that enzymes released from white blood cells into the kidney decrease the size of blood clots. This is a general mechanism but could also explain why patients with thrombotic thrombocytopenic purpura, who develop widespread blood clots in many organs, do not usually develop severe kidney failure.

## 1 Introduction

Von Willebrand factor (VWF), the largest circulatory glycoprotein, supports shear stress-associated platelet adhesion and aggregation at sites of vascular injury (Moake, 1986, Sadler, 2005). VWF is synthesized by endothelial cells (Jaffe et al., 1974) and megakaryocytes (Sporn et al., 1985). VWF is secreted from endothelial cells as ultra-large multimers (ULVWF), which are biologically very potent in adhering to platelets thereby leading to thrombus formation (Moake, 1986). To prevent excessive thrombus formation ULVWF multimers are cleaved by a disintegrin-like and metalloproteinase with thrombospondin type-1 motif, 13 (ADAMTS13), a metalloprotease that cleaves VWF at the 1605Tyr-1606Met residues, leading to the formation of 176 and 140 kDa fragments (Dent et al., 1990, Furlan et al., 1996). In addition to ADAMTS13, four leukocyte proteases, elastase, proteinase 3 (PR3), cathepsin G and matrix metalloprotease 9 (MMP9), have been shown to cleave VWF at, or near, the ADAMTS13 cleavage site (Raife et al., 2009). Other enzymes capable of cleaving VWF are thrombin and plasmin (Berkowitz et al., 1987, Tersteeg et al., 2014, Wohner et al., 2012).

Deficient ADAMTS13 activity leads to thrombotic thrombocytopenic purpura (TTP), which is either congenital (due to mutations) (Levy et al., 2001) or acquired (due to inhibitory antibodies) (Tsai and Lian, 1998). Severe ADAMTS13 deficiency leads to formation of the pathological lesion termed thrombotic microangiopathy (TMA) (Benz and Amann, 2009) which affects renal function due to occluded glomerular capillaries containing VWF-rich microthrombi (Manea et al., 2007). Patients with congenital TTP usually develop renal manifestations, but not renal failure, during acute episodes of disease (Rurali et al., 2015), suggesting that other enzymes may be capable of cleaving VWF in the kidney. These patients may not necessarily present in the neonatal period (Karpman et al., 1996) and may even present as adults, implying that the mutation and residual ADAMTS13 activity may affect the disease phenotype (Camilleri et al., 2008, Lotta et al., 2012, Rurali et al., 2015) and that other proteases may substitute for ADAMTS13 and contribute to VWF cleavage during vascular injury and/or inflammation.

The glomerular basement membrane (GBM) is a ribbon-like specialized extracellular matrix located between the glomerular fenestrated endothelium and podocytes involved in the ultrafiltration of plasma as a barrier to macromolecules (Haraldsson et al., 2008). Components of the GBM are contributed by both podocytes and glomerular endothelial cells (Menon et al., 2012, Striker and Smuckler, 1970), which may explain the presence of ADAMTS13 in the GBM, demonstrated by immune-electron microscopy (Manea et al., 2007), as both podocytes and glomerular endothelial cells express and release ADAMTS13 (Manea et al., 2007, Tati et al., 2011).

When the endothelium is damaged the subendothelium is exposed to blood flow. This will allow the immobilization of platelets to collagen, VWF and fibrinogen in the subendothelium, resulting in platelet adhesion and aggregation (Ruggeri et al., 1999). Under high shear stress ULVWF multimers unfold enabling cleavage to occur thus regulating the size of thrombi by cleaving VWF to smaller multimers (Dong et al., 2002). Such a process would be expected to occur at the interface of the GBM and the glomerular endothelium when glomerular capillaries are damaged. In this study we investigated if VWF is cleaved by components of the GBM and which proteases contribute to cleavage, as proteolytic activity in the GBM could reflect processes occurring in the glomerular subendothelium.

## 2 Methods

### 2.1 Glomerular Basement Membranes

Three preparations of human GBMs, termed GBM-I, GBM-II and GBM-III were used in this study. GBM-I was isolated from post-mortem specimens taken from individuals without renal disease, as previously described (Langeveld et al., 1981, Wieslander et al., 1984), pooled and kept frozen at − 20 °C in lyophilized form after which it was re-suspended in phosphate buffer saline (PBS, Medicago AB, Uppsala, Sweden) for the experiments described below. GBM-II and GBM-III samples were isolated from human cadaver donor kidneys not suitable for transplantation, as previously described (Joosten et al., 2005) using a method developed in rat GBM (Joosten et al., 2002). Membrane fragments were digested with collagenase type Ia and kept in suspension at − 20 °C.

### 2.2 Demonstration of ADAMTS13 and VWF in the GBM by Immunoblotting

The GBM samples were diluted 1:2 under reducing conditions with 2 × sample buffer (0.125 M Tris, 20% glycerol, 4% w/v sodium dodecyl sulfate (SDS), 2% mercapto-ethanol (all from Sigma-Aldrich, St Louis, MO) and 0.001% bromophenol blue (LKB Products AB, Bromma, Sweden)) and subject to sodium dodecyl sulfate-polyacrylamide gel electrophoresis (SDS-PAGE) followed by immunoblotting, as previously described (Tati et al., 2011). ADAMTS13 was detected with goat anti-human ADAMTS13 antibody (BL156, 1:500, Bethyl Laboratories, Montgomery, TX) and, as the secondary antibody, rabbit anti-goat IgG:HRP (Dako, Glostrup, Denmark) at 1:2000. Purified recombinant ADAMTS13 (rADAMTS13), produced in-house (Manea et al., 2010, Tati et al., 2011), diluted 1:50 in the above-mentioned buffer, was used as the positive control. The signal was detected by chemiluminescence using ECL plus (Amersham Biosciences, Uppsala, Sweden).

Von Willebrand factor (VWF) was detected by immunoblotting as above using a combination of pooled mouse anti-human VWF antibodies (specific for the 176 kDa fragment) at 1:500 and mouse M13 anti-human VWF antibody (specific for 140 kDa fragment) at 1:500 (gifts from Prof. Zaverio Ruggeri, The Scripps Research Institute, La Jolla, CA) (Dent et al., 1990). Goat anti-mouse IgG:HRP (Dako) at 1:2000 was the secondary antibody. Plasma purified VWF (L.F.B., Les Ulis, France) at 0.05 U/mL was used as the positive control.

### 2.3 Detection of VWF, ADAMTS13, Elastase, PR3, Cathepsin G and MMP9 in the GBM

The level of endogenous VWF in the GBM was measured by enzyme-linked immunosorbent assay (ELISA) as previously described (Lanke et al., 2008). Levels of ADAMTS13 (Cloud-Clone Corp, Houston, TX), elastase (Hycult Biotech, Uden, Holland), cathepsin G (Nordic BioSite, Täby, Sweden) and MMP9 (R&D Systems, Minneapolis, MN) were measured by ELISA kits according to the manufacturers' protocols. The level of PR3 was measured by ELISA as previously described (Kahn et al., 2009).

### 2.4 VWF Cleavage Detected by Multimer Structure Analysis

VWF cleavage was visualized by VWF multimer structure analysis as previously described (Furlan et al., 1996, Hellström-Westas et al., 2005). GBM samples were diluted 1:50 in buffer (10 mM Tris, 150 mM NaCl, pH 7.4, both from Sigma-Aldrich). Plasma purified VWF at 2 U/mL was added to certain samples. Recombinant VWF (Sinobiological, Beijing, China) lacked ultra-large von Willebrand factor multimers and could not be used in these experiments. The suspensions were dialyzed against 1.5 M urea, 5 mM Tris pH 8.3 (both from Sigma-Aldrich) for 18 h at 37 °C. The samples were run on SDS-agarose gel electrophoresis under non-reducing conditions and VWF multimers were detected by immunoblotting, as previously described (Hellstrom-Westas et al., 2005). Mouse anti-human VWF antibody (1:2900, Dako) was used as the primary antibody and alkaline phosphatase (AP)-conjugated rabbit anti-mouse IgG (1:1500, Dako) or goat anti-mouse IgG:HRP (1:2000, Dako) were used as the secondary antibodies. Detection was carried out by a 5-bromo-4-chloro-3-indolyl-phosphate kit (Bio-Rad Laboratories, Hercules, CA) or by chemiluminescence using ECL plus. Buffer alone was used as the negative control. rADAMTS13 (1:10) added to VWF was used as the positive control. Quantification was carried out by counting the number of visible multimers in each lane, carried out by two independent individuals.

Experiments were designed to study which proteases contributed to VWF cleavage. All inhibition assays were performed by preincubation of the GBM with the inhibitors for 30 min at 37 °C before addition of VWF and dialysis of the samples as described above. To evaluate the specificity of ADAMTS13 activity, certain samples were pre-incubated with either 100 M surplus of polyclonal rabbit anti-human ADAMTS13 antibody (SNO357) (Manea et al., 2010), to block ADAMTS13, or with ethylenediaminetetraacetic acid (EDTA, 40 mM, Merck, Darmstadt, Germany), to block all metalloprotease activity including that of MMP9.

Inhibition was also performed in the presence of complete-mini EDTA-free protease inhibitor cocktail (1 ×, Roche Diagnostics, Mannheim, Germany) to block serine and cysteine proteases, and E64 (10 μM, Sigma-Aldrich) to block cysteine proteases. In certain experiments elastase inhibitor IV (100 μM, Calbiochem, Darmstadt, Germany) was added during the preincubation. To block PR3 activity, the GBM was pre-incubated with rabbit anti-human PR3, previously shown to neutralize PR3 activity (Kahn et al., 2009), at a 100 M surplus. The specificity of the neutralizing activity of the anti-PR3 antibody for PR3 was confirmed using EnzChek peptidase/Protease assay kit (Thermo Fisher Scientific, Waltham, MA). Using the same assay the elastase inhibitor (100 μM) also inhibited PR3 (34 nM) but the PR3-antibody did not inhibit elastase. To block cathepsin G activity the GBM was incubated with a cathepsin G inhibitor (760 μM, Z-Gly-Leu-Phe-chloromethyl ketone, Sigma). The capacity of the individual protease inhibitors used to inhibit their specific protease was confirmed within each experimental setting by immunoblotting or by mass spectrometry, as described below.

### 2.5 Detection of VWF Cleavage Products by Immunoblotting

Protease activity was further tested by detection of the 176 kDa and 140 kDa VWF fragments obtained after ADAMTS13 cleavage at the 1605Tyr-1606Met residues in the A2 domain of VWF. Dialyzed samples were run on SDS-PAGE and immunoblotting was performed using the combination of pooled mouse anti-human VWF antibodies (specific for the 176 and 140 kDa fragments) described above.

### 2.6 FRETS-VWF73 Cleavage Detected by Mass Spectrometry

VWF cleavage activity was further analyzed by mass spectrometry as previously described using a synthetic 73-amino-acid peptide (VWF73) (Kokame et al., 2005, Raife et al., 2009). GBM samples (all diluted to an equivalent elastase concentration of 10 nM) were incubated with FRETS-VWF73 (2 μM final concentration, PeptaNova GmbH, Sandhausen, Germany) in Bis-Tris 5 mM, CaCl_2_ 25 mM, Tween-20 0.005%, pH 6.0 (all from Sigma-Aldrich) for 60 min at 30 °C. Neutrophil elastase (10 nM, Sigma-Aldrich), PR3 (10 nM, Euro Diagnostica, Malmö, Sweden) or cathepsin G (10 nM, Sigma-Aldrich) or rADAMTS13 (10 nM, R&D Systems) were included to indicate the molecular masses expected during cleavage. In certain experiments elastase inhibitor (287 μM), anti-PR3 antibody (100 M surplus), cathepsin G inhibitor (1 mM) or EDTA (40 mM) were pre-incubated with the samples for 60 min at 30 °C. FRETS-VWF73 substrate incubated in Bis-Tris buffer alone served as the negative control. Cleavage products of FRETS-VWF73 substrate were analyzed using matrix-assisted laser desorption ionization-time of flight (MALDI-TOF) mass spectrometry (MALDI Micro MX, Micromass MS Technologies, Manchester, UK).

### 2.7 Perfusion Experiments for the Detection of VWF Cleavage Activity in the GBM

VWF cleavage activity on the surface of endothelial cells was studied using a VenaFlux semiautomated microfluidic platform (Vena8 Endothelial + biochip; Cellix, Dublin, Ireland) as previously described (Tati et al., 2013), with some modifications. Primary glomerular endothelial cells (PGECs, Cell Systems, Kirkland, WA) were pre-stimulated with histamine (200 μM, Sigma-Aldrich) at a shear stress of 5 dynes/cm^2^ for 10 min to trigger VWF release. Platelet-poor plasma (PPP) samples from two previously described TTP patients (patients 5 and 9 in) (Tati et al., 2013) were combined with washed platelets (150–250 × 10^6^/mL from two healthy donors) and perfused over the PGECs at a shear stress of 5 dynes/cm^2^ for 5 min. In some experiments washed platelets were perfused with PBS at the same volume (without PPP) at the same shear stress. To induce cleavage of VWF-platelet strings, GBM samples or neutrophil elastase (25 ng/mL) or purified rADAMTS13 (1:25, as a positive control) were perfused under the same conditions. In certain experiments elastase-inhibitor (287 μM) was added just before perfusion. Quantification was carried out by counting the number of visible VWF-platelet strings in each image (0.6 × 0.8 mm), carried out by two independent individuals.

### 2.8 Human Renal Tissue

Control renal tissue was available from nephrectomized kidney from an adult male patient with renal cancer and was taken from an unaffected area.

### 2.9 Ethics Statement

Blood samples from patients and healthy adult volunteers were taken with the informed written consent of the patients, their parents and the controls. Blood and tissue samples were taken with the approval of the Regional Ethics Board at Lund University. The renal sample was obtained with oral consent and unidentified as per the IRB permit.

### 2.10 Visualization of Neutrophil Elastase in the Human Glomerulus

The presence of elastase in the human renal tissue was analyzed by transmission electron microscopy as described previously (Ståhl et al., 2015). Rabbit anti-human elastase antibody or mouse anti-human CD66 (for detection of neutrophils, Santa Cruz Biotechnology), both at 1:25, were used as primary antibodies. Rabbit IgG or mouse IgG (both from BioLegend) were used as a negative control antibodies. Gold-conjugated goat anti-rabbit IgG: 10 nm or goat anti-mouse IgG: 20 nm (BBI, Cardiff, UK), at 1:100 were used as secondary antibodies. Specimens were observed in a Philips/FEI CM100 transmission electron microscope (Philips, Eindhoven, Holland) operated at 80 kV accelerating voltage and images were recorded with a side-mounted Olympus Veleta camera (Olympus, Münster, Germany) with a resolution of 2048 × 2048 pixels.

### 2.11 Statistics

Differences between the TTP plasma, with and without elastase, were assayed by the two-tailed Mann-Whitney *U* test, and differences between all other samples, with and without inhibitors, by the Kruskal-Wallis multiple comparison test, followed by comparison between specific groups using the Dunn procedure. A p value ≤ 0.05 was considered significant. Statistical analysis was performed using Prism version 7 (GraphPad, La Jolla, CA).

## 3 Results

### 3.1 VWF-cleavage Activity in the GBM

**Fig. 1:**
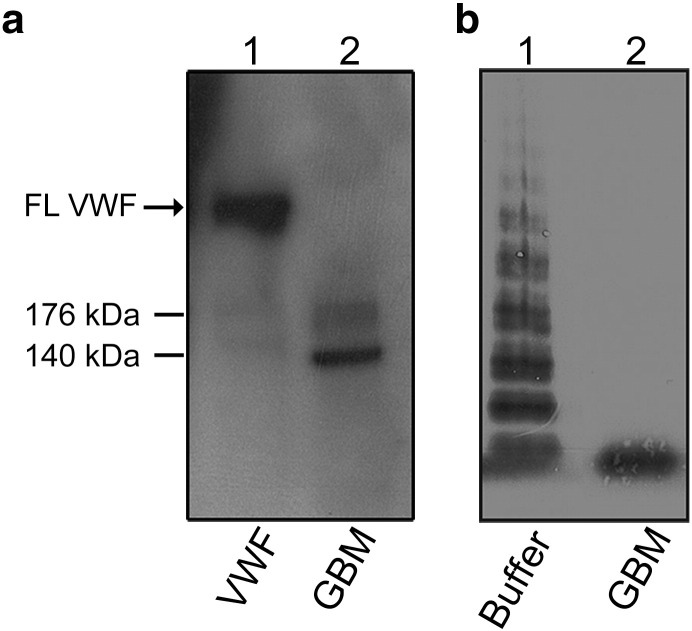
**VWF cleavage activity in the GBM**. (**a**) Immunoblotting exhibited the presence of endogenous VWF in the GBM. Purified VWF was used as the positive control and showed a band representing full-length VWF (FL VWF) depicted by an arrow (lane 1). The GBM sample (GBM-I diluted 1:2) showed cleavage fragments of VWF at approximately 170 kDa and 140 kDa. Reproducible results were obtained from four separate experiments. (**b**) VWF cleavage by the GBM was investigated by VWF multimer structure analysis. The negative control buffer incubated with exogenous VWF showed VWF multimers (lane 1) whereas, GBM-I incubated with exogenous VWF resulted in complete cleavage to VWF dimers (lane 2). Reproducible results were obtained from five separate experiments.

VWF was demonstrated in the GBM. By immunoblotting the positive control (plasma VWF) exhibited a band representing the full-length VWF at approximately 270 kDa (Fig. 1A, lane 1). The GBM sample showed two endogenous VWF cleavage fragments at approximately 170 kDa and 140 kDa (lane 2) indicating that the VWF present in the GBM was already cleaved. The level of VWF in GBM-I was 0.5 μg/mL, as detected by ELISA but below the detection limit in GBM-II (not assayed in GBM-III). The normal plasma value of VWF is 10 μg/mL (Thorell and Blomback, 1984). Endogenous VWF was detectable at a GBM dilution of 1:2 (shown in Fig. 1A) but not at 1:50 (data not shown), the latter dilution was used in all the following experiments to which exogenous VWF was added.

VWF cleavage activity in the GBM sample was demonstrated by VWF multimer structure analysis. The negative buffer control did not show any cleavage of added exogenous VWF (Fig. 1B, lane 1). When exogenous VWF was added to the GBM total VWF cleavage to the smallest multimers was demonstrated (Fig. 1B, lane 2).

### 3.2 Demonstration of ADAMTS13, Elastase, PR3, Cathepsin G and MMP9 in the GBM

ADAMTS13, elastase, MMP9, PR3 and cathepsin G have been shown to cleave VWF (Raife et al., 2009). All proteases were detectable in the GBM samples tested as presented in Table 1.

**Table 1:** Concentration of proteases in the GBM.

Sample	Protein concentrations (ng/mL)^a^				
	ADAMTS13	Elastase	PR3	Cathepsin G	MMP9
GBM-I	7	1.8	35	1.1	ND
GBM-II	30.5	110	115	15.5	3
GBM-III	20	560	616	34.1	12.5

ND: not detectable (under the detection limit).

a Concentrations of proteases in glomerular basement membrane (GBM) samples measured by ELISA.

### 3.3 Cleavage of VWF in the GBM by Neutrophil Proteases but not by ADAMTS13

**Fig. 2:**
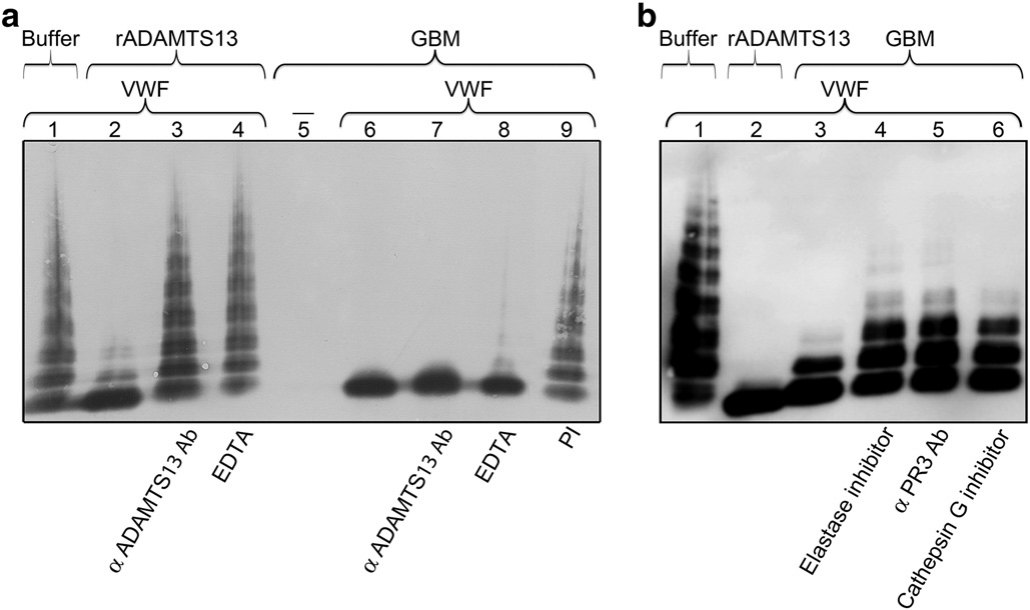
**VWF cleavage by proteases in the GBM detected by multimer structure analysis.** (**a**) The negative control buffer showed VWF multimers (lane 1) and addition of rADAMST13 exhibited cleavage of VWF and the appearance of smaller dimers (lane 2). The cleavage activity of rADAMTS13 was specifically inhibited by either pre-incubation with anti-ADAMTS13 antibody (lane 3) or EDTA (lane 4). GBM-I, without added exogenous VWF, did not exhibit any multimers at a 1:50 dilution (lane 5), whereas incubation with exogenous VWF resulted in complete cleavage to VWF dimers (lane 6). This cleavage activity was not inhibited by pre-incubation with anti-ADAMTS13 antibody (lane 7) but was slightly inhibited by pre-incubation with EDTA (lane 8). This inhibition was more pronounced in the presence of the protease inhibitor cocktail (PI, lane 9). Reproducible results were also obtained from altogether five experiments with GBM-I, two experiments with GBM-II and two experiments with GBM-III (not all inhibitors were tested in each setting, see text). (**b**) Exogenous VWF incubated with control buffer showed VWF multimers (lane 1) and addition of rADAMST13 exhibited cleavage of VWF (lane 2). GBM-III incubated with exogenous VWF resulted in cleavage of VWF (lane 3). This cleavage activity was partially inhibited by pre-incubation with elastase inhibitor (lane 4), anti-PR3 antibody (lane 5) or cathepsin G inhibitor (lane 6). Reproducible results were obtained from three experiments with GBM-III and with GBM-II, respectively.

Experiments were designed to demonstrate if ADAMTS13 within the GBM cleaves VWF. The negative buffer control did not show any cleavage of added exogenous VWF (Fig. 2A, lane 1) which was shown to be cleaved by rADAMTS13 (Fig. 2A, lane 2), the positive control. The specific VWF cleavage activity of rADAMTS13 was inhibited by pre-incubation with both anti-human ADAMTS13 antibody SNO357 (Fig. 2A, lane 3), and EDTA (40 mM, Fig. 2A, lane 4), as expected. GBM alone did not exhibit endogenous VWF at the concentration used (1:50, Fig. 2A, lane 5). When exogenous VWF was added the VWF cleavage activity exerted by the GBM sample (Fig. 2A, lane 6) was not inhibited by the anti-ADAMTS13 antibody alone (Fig. 2A, lane 7) but was slightly inhibited by EDTA (Fig. 2A, lane 8). A comparison was carried out between the number of multimers visible in the buffer (median 10, range 10–15, n = 9 experiments) with multimers visible in the presence of GBM (median 1, range 1–4, n = 9, p < 0.0001), in the presence of the GBM with added anti-ADAMTS13 antibody (median 2, range 1–3, n = 4, p < 0.01) or with added EDTA (median 2, range 1–6, n = 6, p < 0.01) suggesting minimal contribution of a metalloprotease, which was not ADAMTS13.

The possibility that a serine or cysteine protease induced VWF cleavage by the GBM was investigated. The protease inhibitor cocktail incubated with the GBM in the presence of exogenous VWF induced almost complete inhibition (Fig. 2A, lane 9, median number of multimers 6, range 3–10, n = 5 experiments, non-significant compared to buffer alone) suggesting that cleavage was mostly achieved by a serine and/or cysteine protease. The cysteine protease inhibitor E64 pre-incubated with the GBM did not inhibit VWF cleavage (data not shown). Pre-incubation of E64 in combination with the protease inhibitor cocktail did not increase the inhibition induced by the protease inhibitor cocktail alone (data not shown). Thus cysteine proteases did not contribute to VWF cleavage.

The presence of neutrophil proteases in the GBM, demonstrated by ELISA (Table 1) and the finding that serine proteases in the GBM cleaved VWF (with a minimal contribution of a metalloprotease as shown in Fig. 2A, lane 8), led us to focus on the VWF-cleaving activity of serine proteases within the GBM. Pre-incubation of GBM samples with the elastase inhibitor, anti-PR3 antibody and cathepsin G inhibitor all showed partial inhibition of VWF cleavage activity (Fig. 2B, lanes 4–6, respectively) with somewhat stronger inhibition when elastase and PR3 were blocked compared to cathepsin G.

**Fig. 3:**
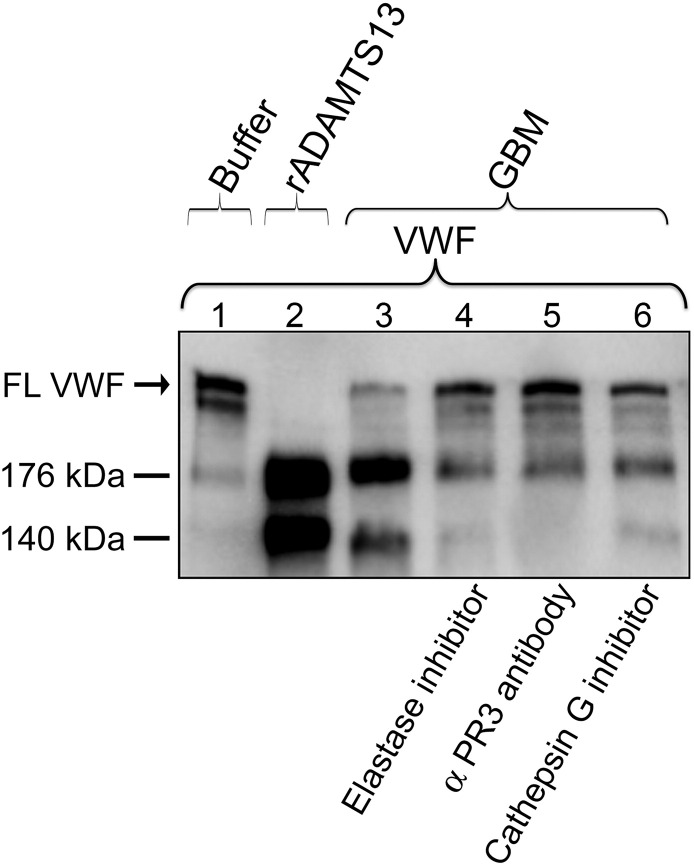
**VWF cleavage induced by neutrophil proteases in the GBM detected by immunoblotting.** Immunoblotting was performed to investigate VWF cleavage by the GBM. The negative control buffer alone showed full-length VWF (FL VWF) along with a weak cleavage product at 176 kDa (lane 1). rADAMTS13 induced VWF cleavage by exhibiting the two cleavage products at 176 kDa and 140 kDa (lane 2). GBM-III incubated with exogenous VWF showed VWF cleavage demonstrated by the appearance of two cleavage products at 176 kDa and 140 kDa (lane 3). Pre-incubation with elastase inhibitor or anti-PR3 antibody exhibited inhibition with a weak band at 176 kDa (lanes 4 and 5, respectively). Pre-incubation with the cathepsin G inhibitor also showed similar but lesser inhibition (lane 6). Reproducible results were obtained from four experiments with GBM-II and two experiments with GBM-III.

VWF cleavage activity was further investigated by immunoblotting using antibodies against the cleavage products. The negative buffer control showed full-length VWF and a faint band corresponding to 176 kDa band (Fig. 3, lane 1). The positive control, VWF exposed to rADAMTS13, showed specific cleavage fragments of 176 and 140 kDa (Fig. 3, lane 2). VWF exposed to the GBM exhibited two similar cleavage products corresponding to 176 kDa and 140 kDa (Fig. 3, lane 3). Elastase inhibitor, anti-PR3 antibody and cathepsin G inhibitor induced inhibition (Fig. 3, lanes 4–6 respectively), more pronounced when elastase and PR3 were inhibited.

### 3.4 Cleavage of FRETS-VWF73 by the GBM

**Fig. 4:**
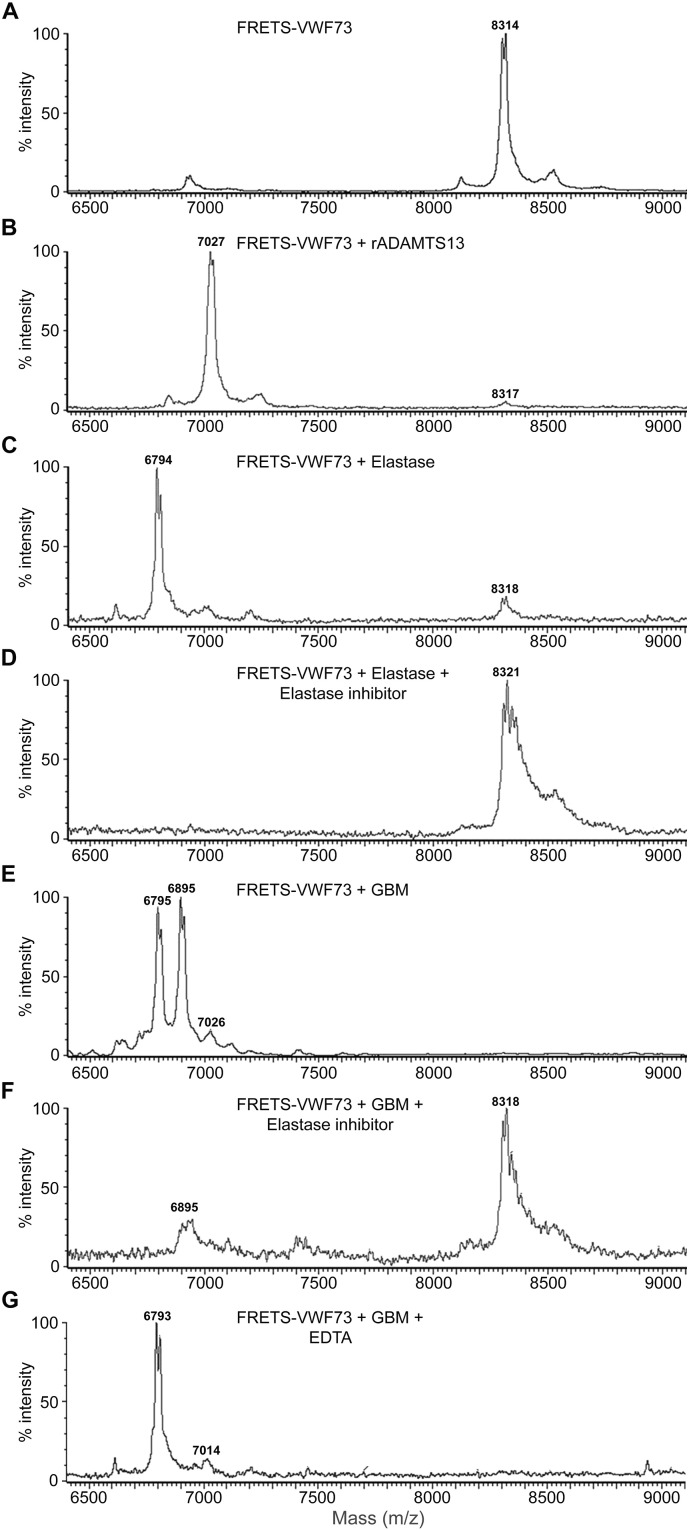
**Cleavage of FRETS-VWF73 detected by mass spectrometry.** MALDI-TOF mass spectrometry was performed to analyze VWF cleavage activity of GBM samples using FRETS-VWF73. A. FRETS-VWF73 incubated with buffer alone served as the negative control showing a peak at molecular mass 8314 Da. B. FRETS-VWF73 incubated with rADAMTS13 exhibited a peak at 7027 Da. C. FRETS-VWF73 incubated with neutrophil elastase exhibited a fragment peak at 6794 Da. D. FRETS-VWF73 incubated with neutrophil elastase and pre-incubated with the elastase inhibitor abolished elastase-induced cleavage. E. GBM-III incubated with FRETS-VWF73 generated two major cleavage fragments corresponding to 6795 Da (elastase/PR3-cleavage) and 6895 Da (MMP9-cleavage) and a minor fragment at 7026 Da (cathepsin G/ADAMTS13-cleavage). F. GBM-III as in (E) pre-incubated with the elastase inhibitor markedly abolished the cleavage with a minor fragment remaining at 6895 Da. G. GBM-III pre-incubated with EDTA abolished MMP9-induced cleavage but not that induced either by elastase/PR3 (6793 Da) or by cathepsin G (7014 Da). Reproducible results were obtained from both GBM-II and GBM-III from three separate experiments.

To demonstrate VWF cleavage, GBM samples incubated with fluorescence resonance energy transfer (FRETS)-VWF73 substrate were analyzed by matrix-assisted laser desorption ionization-time of flight (MALDI-TOF) mass spectrometry. A negative control buffer showed uncleaved FRETS-VWF73 at a molecular mass of 8314 Da (Fig. 4A). rADAMTS13 cleavage of FRETS-VWF73 generated a peak at 7027 Da (Fig. 4B). Elastase-induced cleavage of FRETS-VWF73 yielded a fragment of 6794 Da (Fig. 4C). This is consistent with the previously reported molecular mass (Raife et al., 2009). Pre-incubation with the elastase inhibitor abolished elastase-induced cleavage (Fig. 4D). Similar results were obtained using cathepsin G and its inhibitor (data not shown).

GBM incubated with FRETS-VWF exhibited two major fragments with molecular masses of 6795 and 6895 Da and a minor fragment of 7026 Da (Fig. 4E) corresponding to cleavage by elastase/PR3, MMP9, and ADAMTS13/cathepsin G, respectively, as described (Raife et al., 2009). Pre-incubation with the elastase inhibitor almost completely abolished the cleavage suggesting a predominant role of elastase (or PR3 as the inhibitor blocked both proteases at the concentration used). A fragment with a mass of 6895 Da remained, corresponding to cleavage by MMP9 (Fig. 4F). Pre-incubation with EDTA abolished the 6895 Da fragment (induced by MMP9). A major fragment with a mass of 6793 Da and a minor fragment of 7014 Da remained, corresponding to cleavage by elastase/PR3 and cathepsin G, respectively (Fig. 4G). Taken together, these data suggest that elastase and/or PR3 within the GBM cleave VWF, with a minor role of cathepsin G and MMP9. The following experiments specifically addressed the role of elastase in GBM-mediated VWF cleavage.

### 3.5 Elastase in the GBM Cleaves VWF-platelet Strings on the Surface of Endothelial Cells

**Fig. 5:**
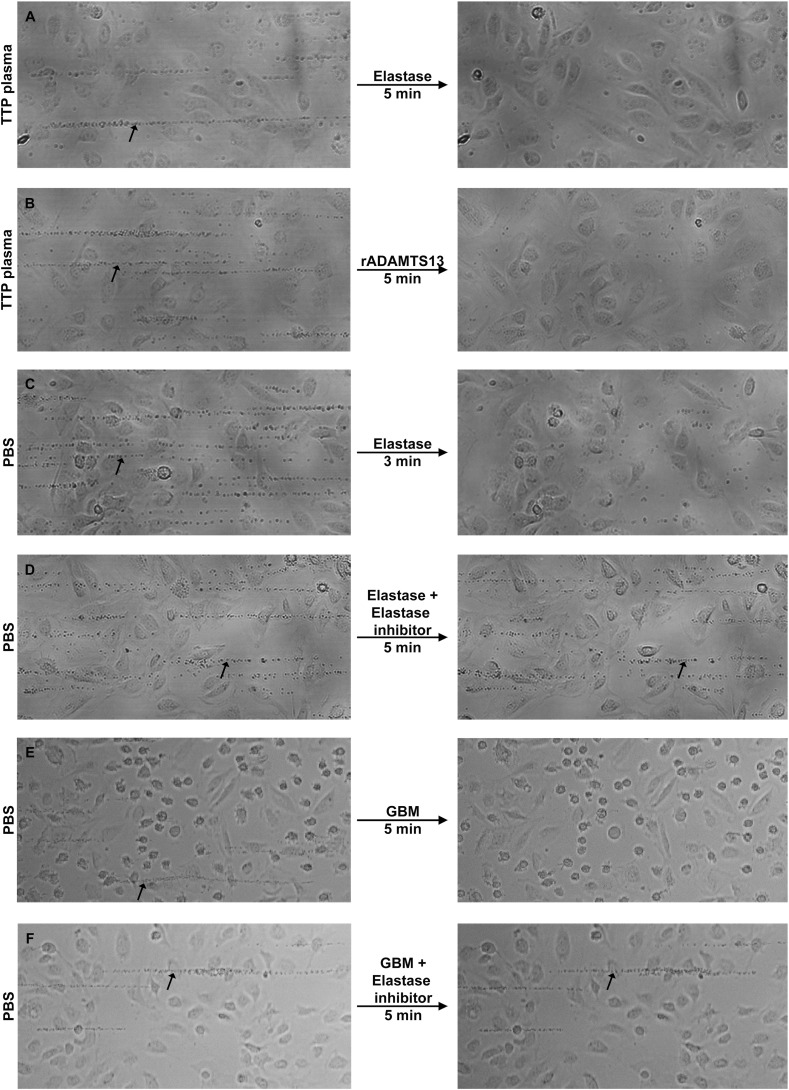
**GBM cleaves VWF on the surface of the glomerular endothelial cell.** VWF-platelet strings were visualized by perfusion of platelets over primary glomerular endothelial cells (PGECs). A. Perfusion of PPP from a TTP patient (Patient 9 in (Tati et al., 2013)) combined with normal washed platelets onto histamine-stimulated PGECs led to the formation of VWF-platelet strings (see arrow in left panel). Neutrophil elastase perfused onto the cells cleared the strings at the same locations within 5 min (right panel). B. Perfusion of PPP from the same TTP patient over PGECs as in A (arrow in left panel indicates VWF-platelet strings) and rADAMTS13 cleared the VWF-platelet strings (right panel). C. Normal washed platelets in phosphate buffer saline (PBS, without plasma) generated VWF-platelet strings on the cell surface (arrow in left panel) and were cleared upon addition of neutrophil elastase (right panel). D–F. Normal washed platelets in PBS (without plasma) generated VWF-platelet strings on the cell surface (arrows in left panels), same conditions as in C. Upon pre-incubation with elastase inhibitor IV, cleavage activity of neutrophil elastase was abolished and strings were visible (arrow in right panel D). Similarly strings were cleared upon perfusion of GBM-III (right panel E) and pre-incubation with the elastase inhibitor abolished the cleavage (arrow in right panel F). Images are representative of three separate experiments with reproducible results from GBM-II and GBM-III. All images were acquired using a Carl Zeiss Axiovert 40CFL microscope (Carl Zeiss, Jena, Germany) equipped with a digital camera DP 450 (Deltapix, Maalov, Denmark), using a Plan Apochromat 20 × objective.

Perfusion experiments were carried out to detect the formation and cleavage of VWF-platelet strings on primary glomerular endothelial cells (PGECs). Perfusion of TTP patients' platelet-poor plasma (PPP, n = 2 patients) combined with normal washed platelets onto histamine-stimulated PGECs exhibited stable VWF-platelet strings (Fig. 5A and B, left panels). Neutrophil elastase (Fig. 5A, right panel) or rADAMTS13 (Fig. 5B, right panel) perfused onto these strings led to clearance of the strings within 5 min. Quantification was carried out demonstrating that the number of VWF-platelet strings formed in the presence of TTP plasma was 5–45 strings, median 30 (n = 5 experiments) and when elastase was added the median and range decreased to 0 strings (n = 4) p < 0.05.

Similarly, normal washed platelets without PPP perfused onto histamine-stimulated PGECs exhibited stable VWF-platelet strings (Fig. 5C–F, left panels), which were cleared within 3 min upon perfusion of neutrophil elastase (Fig. 5C, right panel). In the presence of the elastase inhibitor the cleavage of VWF-platelet strings, induced by elastase, was abolished (Fig. 5D, right panel). Similarly, perfusion of GBM samples also cleared VWF-platelet strings within 5 min (Fig. 5E, right panel), an effect inhibited by the elastase inhibitor (Fig. 5F, right panel). Quantification showed the formation of VWF-platelet strings in the presence of PBS (2–50 strings, median 19, n = 13 experiments) compared to the same samples in the presence of elastase (0 strings, median and range, n = 3, p < 0.05). In the presence of elastase inhibitor strings were formed (range 7–40 strings, median 40, n = 3, non-significant compared to PBS buffer). In the presence of GBM fewer strings were visible (range 0–1, median 0, n = 4, p < 0.05) an effect totally inhibited in the presence of the elastase inhibitor (range 2–14, median 12, n = 3, non-significant compared to PBS).

### 3.6 Elastase is Released Into the GBM in Proximity of Leukocytes

**Fig. 6:**
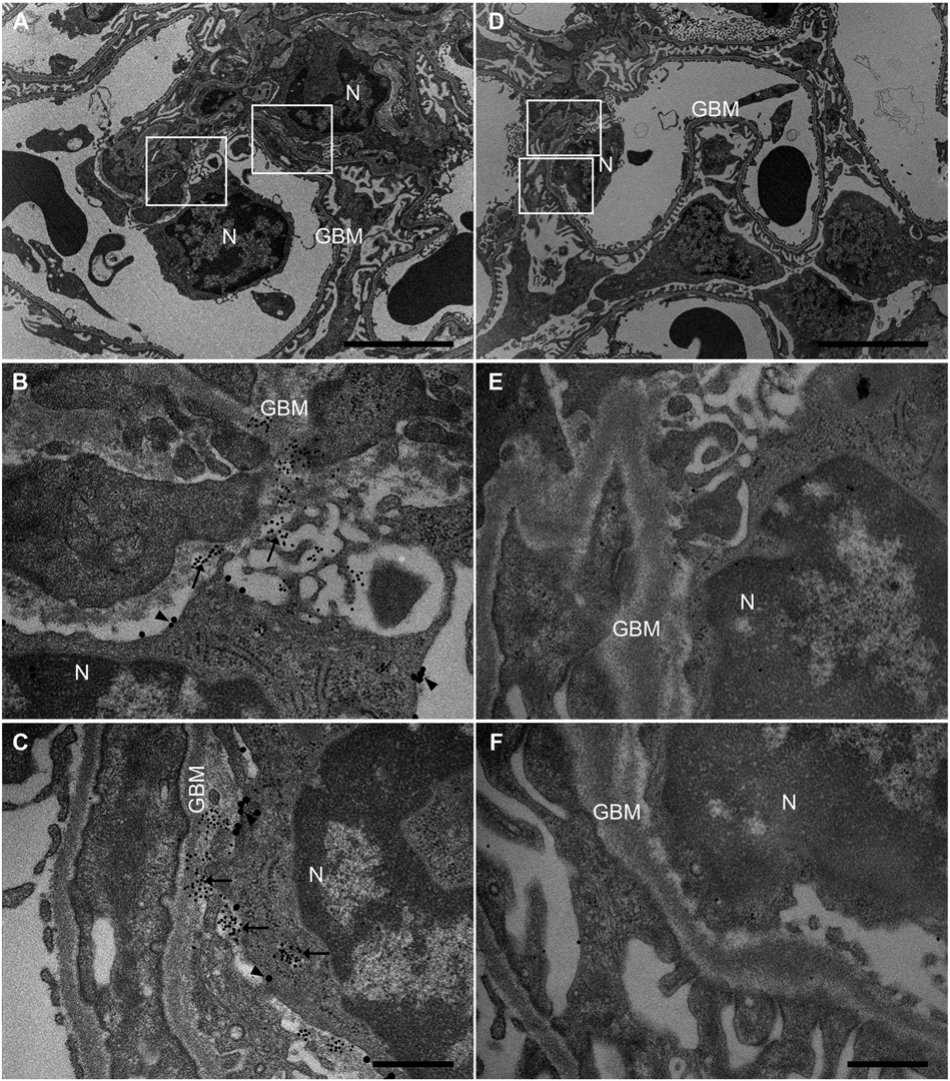
**Electron microscopy to detect elastase in renal tissue**. The presence of elastase in the glomerulus was examined by electron microscopy. A. Ultramorphology showing an overview from a glomerulus: neutrophil (N) and glomerular basement membrane (GBM). Boxed areas are enlarged in panels B and C. B and C. Renal cortex, at higher magnification, labeled with anti-elastase (10 nm, arrows) and with anti-CD66 (20 nm, arrowheads) to label neutrophils. D. Ultramorphology showing an overview from a glomerulus: neutrophil (N) and glomerular basement membrane (GBM). Boxed areas are enlarged in panels E and F. E and F. Control antibodies showed weak or no signal in the renal tissue. Samples were examined in a JEOL JEM 1230 transmission electron microscope (JEOL, Peabody, Mass., USA) at 60 kV accelerating voltage. Images were recorded with a Gatan Multiscan 791 CCD camera using DigitalMicrograph™ software. Scale bar: 5 μm (A and D) and 500 nm (B, C, E and F).

Ultramorphology of kidney sections from unaffected areas of a resected cancer kidney exhibited elastase (using gold-conjugated anti-elastase antibody) within and in proximity of neutrophils (detected by gold-conjugated anti-CD66) in glomerular capillaries. Elastase is released from granules onto the glomerular endothelium and into the GBM (Fig. 6). This indicates that the source of neutrophil elastase within the GBM is most probably neutrophils in the glomerular capillaries.

## 4 Discussion

Neutrophil proteases were previously shown to cleave VWF (Raife et al., 2009), but the physiological setting in which this may occur is unknown. Here we show that neutrophil proteases, released into the GBM from neutrophils within glomerular capillaries, cleave VWF and may thus protect the glomerular subendothelium from thrombotic damage induced by the adherence of platelets to ULVWF multimers. ADAMTS13, the known physiological VWF protease, as well as neutrophil-derived elastase, PR3, cathepsin G and MMP9 were all present in the GBM preparations, in which we could demonstrate a major role for elastase and/or PR3 in VWF cleavage. Neutrophil proteases may thereby function as VWF-cleaving proteases in the glomerular subendothelium and prevent the formation of microthrombi. This effect would be pertinent at times of infection or inflammation, when the endothelium is activated and neutrophils infiltrate the glomeruli.

In the resting state the presence of neutrophils in glomeruli would be minimal and thus VWF-cleaving activity by neutrophil proteases negligible. During infection and inflammation neutrophil influx will occur and proteases released into the glomerular capillary lumen will migrate towards the subendothelium, as demonstrated here. Thus, neutrophil proteases may prevent thrombosis from occurring on a thrombogenic vascular surface. In the presence of functional ADAMTS13, released locally from the activated glomerular endothelium (Tati et al., 2011) or circulating in the plasma, a combined anti-thrombotic effect would be achieved. In addition to proteases, neutrophils may also secrete peptides that regulate the anti-thrombotic activity of ADAMTS13 (Pillai et al., 2016).

Elevated neutrophil elastase occurring during infectious episodes (Mikes et al., 2014) could explain why certain TTP patients are partially protected and remain asymptomatic for long periods in spite of ADAMTS13 deficiency. TTP recurrences are often triggered by infections during which neutrophils infiltrate tissue. Although lack of ADAMTS13 predisposes the endothelium to a prothrombotic state the presence of proteases released by neutrophils could offer partial protection. VWF cleavage mediated by neutrophil proteases could prevent the fulminant formation of microthrombi. In spite of development of thrombotic microangiopathy many TTP patients do not develop renal failure, which could be due to the phenotype of their mutation (Rurali et al., 2015), as well as neutrophil protease activity in glomeruli.

The main inhibitor of elastase and PR3 in plasma is α1-antitrypsin (Ohlsson, 1971). The VWF-cleaving activity of neutrophil elastase, demonstrated under flow conditions, was maintained after PGECs were in contact with TTP plasma (lacking ADAMTS13 activity). The presence of an elastase inhibitor would most probably be minimal under the conditions used, as the plasma had been washed away in the perfusion process. In the glomerular subendothelium and the GBM the concentration of plasma would be very low and thus elastase-induced VWF cleavage could proceed in an uninhibited manner and prevent formation of large VWF-platelet thrombi thereby protecting the kidney from fulminant damage during mild inflammation. The effects of neutrophil proteases are most probably related to their local concentrations. During massive neutrophil-mediated inflammation excessive release of neutrophil proteases will occur and incur injury due to the destructive properties of these proteases.

Cultured endothelial cells from umbilical veins and the glomerular microvasculature release ULVWF (Dong et al., 2002). The release of VWF strings from histamine-stimulated PGECs to which platelets bound, confirmed this finding in this study. Elastase alone, and within the GBM, was shown to cleave the VWF-platelet strings, as was also demonstrated for rADAMTS13. ADAMTS13 cleaves VWF at the Y1605-M1606 bond resulting in 176 and 140 kDa fragments (Dent et al., 1990, Furlan et al., 1996). Similarly, elastase and PR3 were shown to cleave VWF at the V1607-T1608 bond, cathepsin G at Y1605-M1606 and MMP9 at the M1606-V1607 cleavage site (Raife et al., 2009). GBM samples incubated with exogenous VWF generated similar cleavage fragments in the current study. The findings suggest that neutrophil proteases cleave large VWF multimers and could do so within the glomerulus.

The GBM possesses a unique position between the fenestrated glomerular endothelium and podocytes allowing it to function as part of the filtration barrier and as a recipient of factors secreted from both adjacent cells. Interestingly, ADAMTS13 is present within the GBM, as we have demonstrated previously (Manea et al., 2007) and in the current study, but apparently does not contribute to VWF cleavage. ADAMTS13 is a 190 kDa protein synthesized in both glomerular endothelial cells (Tati et al., 2011) and podocytes (Manea et al., 2007) both of which contribute to the constitution of the GBM. ADAMTS13 in the GBM would most probably originate from podocytes, as secretion from glomerular endothelial cells is apical (Shang et al., 2006). Podocyte-derived growth factors such as vascular endothelial-derived factor and angiopoietin-1 have been shown to affect glomerular endothelial cells (Eremina et al., 2006, Fogo and Kon, 2010, Haraldsson and Nystrom, 2012, Satchell et al., 2004). Interactions between podocyte-derived factors and the glomerular endothelium have been suggested to occur by transfer of substances counter to the direction of filtration via sub-podocyte spaces (Fogo and Kon, 2010, Salmon et al., 2009). Thus the ADAMTS13 present in the GBM could originate from podocytes although it is unclear if such a large protein could transfer through the GBM and cleave VWF at the GBM-endothelial interface. The lack of ADAMTS13 VWF-cleaving activity in our GBM preparations may be due to inherently low levels in the GBM, alternatively to the process of GBM isolation or the relative concentration of proteins remaining in the GBM after storage. Nevertheless, the results indicate that there are additional VWF-cleaving proteases within the GBM and that these proteases can replace the function of ADAMTS13 at this site.

Elastase is released from azurophilic granules in the neutrophil cytoplasm. Ultramorphology of an area of unaffected tissue in a kidney removed from a patient with renal cancer demonstrated the passage of neutrophils through glomerular capillaries where elastase was released from granules onto the adjacent endothelial cells. Likewise, other neutrophil proteases stored in azurophilic granules, such as PR3 and cathepsin G, would be expected to be concurrently released. An inflammatory process could be expected even in the unaffected portion of the kidney removed due to cancer explaining why neutrophils were present. Similarly, in kidneys removed post-mortem a certain degree of neutrophil influx would be expected. Neutrophils will accumulate and roll on endothelial cells during inflammatory conditions in which endothelial cells are activated and express adhesion molecules (Noris and Remuzzi, 1995). Under these conditions the endothelium may be damaged and the subendothelium will be exposed. We propose that proteases released from neutrophils will thus come in contact with the endothelium and subendothelium and protect these surfaces from excessive platelet-VWF interactions by cleaving VWF multimers. Neutrophil proteases will be taken up by the GBM and protect the endothelial-GBM interface from further injury and capillary occlusion.

In summary, this study shows that neutrophil proteases, taken up or trapped within the GBM, can cleave VWF and thereby offer protection against disproportionate platelet-VWF thrombus formation in the glomerulus, a finding which may explain how the kidney is protected from microthrombi formation in the subendothelium during inflammation and also why some TTP patients do not exhibit severe renal damage in spite of the development of TMA in their kidneys.

## Funding Sources

This study was supported by grants from The Swedish Research Council (K2013-64X-14008-13-5 and K2015-99X-22877-01-6), The Knut and Alice Wallenberg Foundation (Wallenberg Clinical Scholar 2015.0320), The Torsten Söderberg Foundation (MT2/15), Skåne Centre of Excellence in Health, Crown Princess Lovisa's Society for Child Care, Region Skåne and The Konung Gustaf V:s 80-årsfond, The Ingabritt and Arne Lundberg Research Foundation (all to DK), The Royal Physiographic Society (to RT). The funding sources had no role in the writing of the manuscript or the decision to publish.

## Conflicts of Interest

The authors report no conflicts of interest.

## Author Contributions

Ramesh Tati: performed the research, analyzed data and wrote the paper.

Ann-Charlotte Kristoffersson: performed experiments and analyzed data.

Minola Manea Hedström: performed experiments and analyzed data.

Matthias Mörgelin: performed experiments and analyzed data.

Jörgen Wieslander: provided GBM.

Cees van Kooten: provided GBM and assisted in writing the paper.

Diana Karpman: designed the project, analyzed data and wrote the paper.
